# Eco-friendly carbon-nanodot-based fluorescent paints for advanced photocatalytic systems

**DOI:** 10.1038/srep12420

**Published:** 2015-07-23

**Authors:** So Young Park, Hyun Uk Lee, Young-Chul Lee, Saehae Choi, Dae Hyun Cho, Hee Sik Kim, Sunghee Bang, Soonjoo Seo, Soon Chang Lee, Jonghan Won, Byung-Chul Son, Mino Yang, Jouhahn Lee

**Affiliations:** 1 Advanced Nano-Surface Research Group Korea Basic Science Institute (KBSI), Daejeon 305-333, Republic of Korea; 2 Department of BioNano Technology, Gachon University, Gyeonggi-do 461-701, Republic of Korea; 3 Sustainable Bioresource Research Center, Korea Research Institute of Bioscience and Biotechnology (KRIBB), Daejeon 305-806, Republic of Korea; 4 Department of Engineering (Nanotechnology Engineering), University of Waterloo 200 University Avenue West, Waterloo, Ontario, N2L 3G1, Canada; 5 Department of Applied Chemistry and Biological Engineering, Chungnam National University, Daejeon 305-764, Republic of Korea; 6 Korea Advanced Institute of Science and Technology (KAIST), Research Analysis Center, Daejeon 305-701, Republic of Korea; 7 Korea Basic Science Institute (KBSI), Daejeon 305-333, Republic of Korea

## Abstract

Fluorescent carbon nanomaterials, especially zero-dimensional (0D) carbon nanodots (CDs), are widely used in broad biological and optoelectronic applications. CDs have unique characteristics such as strong fluorescence, biocompatibility, sun-light response, and capability of mass-production. Beyond the previous green CD obtained from harmful natural substances, we report a new type of fluid-based fluorescent CD paints (C-paints) derived from polyethylene glycol (PEG; via simple ultrasound irradiation at room temperatures) and produced in quantum yields of up to ~14%. Additionally, C-paints possess a strong, UV- and visible-light-responsive photoluminescent (PL) property. Most especially, C-paints, by incorporation into a photocatalytic system, show additional roles in the emission of fluorescent light for activation of TiO_2_ nanoparticles (NPs) and the resultant detoxification of most organic dyes, thus further enabling embarkation in advanced water purification.

Fluorescent carbon-based nanomaterials with low toxicity and excellent chemical- and photo-stability show high capacity in the fields of environmental engineering and biological imaging as well as in therapeutic procedures^1^,^2^,^3^. Various carbon nanoparticles (NPs) can be utilized as fluorescent sources such as quantum dots^4^, nanospheres^5^, nanoribbons^6^, nanowires^7^ and nanotubes^8^. They are generally prepared by laser ablation^9^, the candle-soot methods^10^, electrochemical oxidation of graphite^11^, microwave pyrolysis of sucrose^12^, or proton-beam irradiation of nanodiamonds^13^. Mass production of fluorescent carbon nanomaterials, however, remains challenging because of non-economic production cost by rare simple production methodologies^14^. It is not surprising, then, that carbon nanomaterials with various textures such as softened or sticky properties, have been explored in diverse biological and environmental fields. The discovery of new types of fluorescent carbon nanoprobes with viscoelastic dynamics and stretchable properties for curved bio-environmental systems and flexible optoelectronics is necessary^15^,^16^.

Recently, fluorescent nanomaterials of viscous type have become important due to their softened properties, good adhesion ability on various materials surfaces and easy-to-process^17^. Compared to solid state, this liquid form of fluorescent carbon has advantages such as facile synthesis method, solvent-free process, transparency and miscible properties^18^. The liquid carbon-rich polymers are scalable in an economic point of view. Of particular interest is the recent finding that viscous-fluorescent CDs can be mixed with various materials to become strongly fluorescent in UV- and visible-spectral regions^19^,^20^,^21^.

Herein, we report a facile one-step fabrication of carbon-based sticky and fluorescent nanodots: namely, CD-based paints (C-paints) derived from polyethylene glycol (PEG) and exhibiting stable photoluminescence (PL) properties under the proper conditions (maximum quantum yield: ~14%). Such C-paints do not require any additive solvent, acid, base, or surface passivation agent in the synthetic process, which leads to toxic-free by-products or side effects. C-paints are suitable for fabrication on CDs with efficient structures and properties, thanks to biocompatible PEG’s low price and eco-friendliness^22^. Previously, our group reported CD fabrication from food waste and harmful green microalgae by modified ultrasound irradiation^23^,^24^. However, the CDs’ relatively low PL intensity and physical states significantly limits their application to various fluorescence-based optoelectronic devices. In the present study, by utilizing CDs characteristics, fluid-based C-paints could be modified to free or soluble in water as a flexible light source as to dissolve paints in water. As a result, we found C-paints to be one of the most effective fluorescent sources for enhancement of photocatalytic reaction on the photocatalysts. We optimized the C-paints/water/TiO_2_ ratio and then compared the activity with bare TiO_2_ NPs under UV- and visible-light. The increasing availability of viscous fluorescent carbon has created widespread interest in their use in water-soluble fluorescent systems for bio-environmental purposes.

## Results

### Morphological structures and size distribution

**Figure 1:**
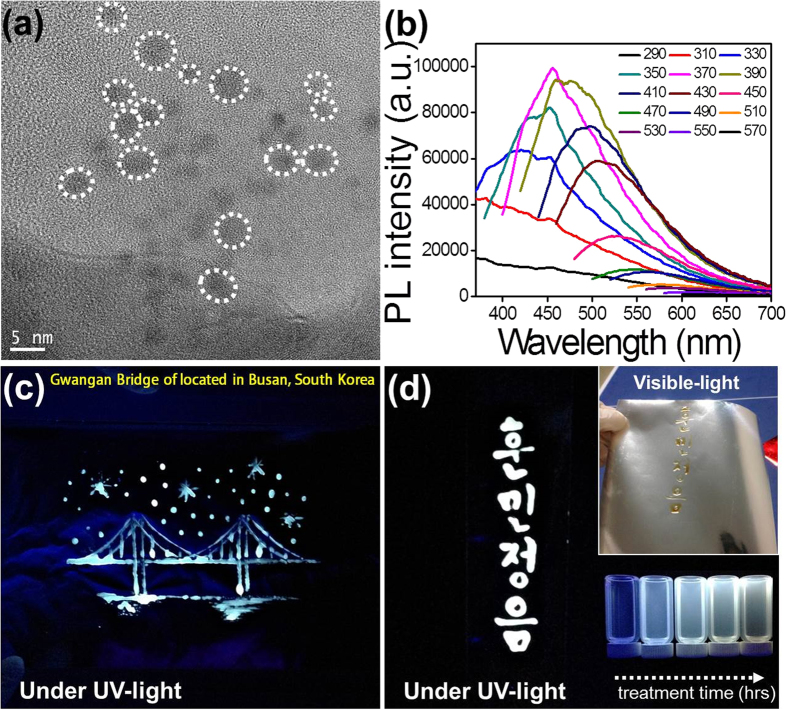
Morphology structures and optical properties of C-paints. (**a**) TEM image of C-paints prepared using our simple method, showing the C-paints are spherical and well dispersed, (**b**) PL emission spectra of C-paints at different excitation wavelengths, (**c**) Digital photographs of C-paints marked fluorescent landscape on aluminum foil, and (**d**) Korean alphabet, Hangul painting captured on silver paper under UV-light (left panel), where the inset is the original text of Hangul (right-top panel) under visible-light and photograph (right-bottom panel) of the samples excited by a UV lamp.

The C-paints were assessed in their morphology and size distribution using transmission electron microscopy (TEM) and atomic force microscopy (AFM). TEM images show that these C-paints are uniform in size ranging from 2 to 6 nm in diameter. AFM images further confirm the uniformity of the C-paints. The height of the C-paints ranges from 1 to 8 nm (overall average: ~4.3 nm), without aggregation (Fig. 1a and Supplementary Fig. 1).

### X-ray diffraction (XRD) patterns

The C-paints were shown to be highly water-soluble as well as particle-aggregation-resistant over several days. The C-paints’ XRD patterns showed broad peaks at 2θ = 21.9° and a weak peak at 2θ = 43.4°, which were assignable to the (002) and (101) diffraction patterns of graphitic carbon, respectively (Supplementary Fig. 2)^23^. These peaks revealed what was almost an amorphous carbon phase, which was attributed to the introduction of oxygen-containing groups by physico-chemical reaction^24^. The former peak corresponds to the interlayer spacing of ~3.8 Å, which is slightly longer than that between the (002) planes in bulk graphite (~3.5 Å)^23^,^24^.

### Fourier transform infrared spectroscopy (FT-IR) and X-ray photoelectron spectra (XPS) analysis

FT-IR spectra were used to investigate the bonding composition and functional groups for the sample of as-synthesized C-paints at different synthesis time (Supplementary Fig. 3). The peak at 1130 cm^−1^ is assigned to a –CH_2_ stretching vibration^23^,^24^,^25^. The absorption at 2850–3000 cm^−1^ is attributed to C-H stretching vibration. The broad peaks at 3200–3600 cm^−1^ indicates O-H bonding vibration^24^, which results in the hydrophilicity of C-paints and improves their stability and dispersibility in water, without any further surface passivation^23^. The chemical bonding states of the functional carbon and oxygen groups of the C-paints were examined by XPS analysis. The XPS spectra exhibited three peaks between 284.6 and 530.6 eV, which arises from C1s and O1s, respectively. The atomic ratio of carbon to oxygen is 2.14. A high-resolution scan of the C1s region showed deconvolution of the C1s peaks present in the carbon atoms’ different functional groups [-C-C or -C-H (284.6 eV), -C-O(H) (286.2 eV), and C-O-C=O (287.3 eV) peaks in the C1s spectrum], as evidenced in Supplementary Fig. 4 and Supplementary Tables 1 and 2^24^,^26^. These functional groups signify the solubility of C-paints in water without further chemical modification. All of the surface components of the C-paints measured using XPS are in good agreement with the FT-IR spectral results. The presence of oxygen-containing functional groups on the surface of amorphous C-paints can increase the surface properties along with the potential for photocatalytic and other flexible optoelectronics applications.

### Optical properties of C-paints

As shown in the UV-Vis spectra in Supplementary Fig. 5, C-paints have one strong absorption band centered at 245 nm and the other broad band found in the visible range^27^. Upon excitation wavelength at 245 nm, bright fluorescence centered at 400 and 500 nm could be observed. Additionally, the emission intensity of the C-paints gradually increased as synthesis time increased. When the PEG polymer was treated by ultrasound irradiation for more than 1 hr, shoulder peaks began to appear in the PL emission spectra, as shown in Fig. 1b. The C-paints, compared with the original polymer, exhibited a sharply increased fluorescence under UV- as well as visible-light. Their emission spectra shifted from UV- to visible-light with the changes in the excitation conditions. Correspondingly, the emission in blue or red was observed at the wavelength ranging from 290 nm to 570 nm, which is a common phenomenon of reported CDs. The different-sized NPs (quantum effect) and/or emissive trap sites on the CD surface or another various mechanisms have been studied in order to establish the multicolor PL dependent on excitation wavelengths^9^,^28^,^29^. As seen in Supplementary Fig. 6, the PL spectra of the C-paints were gradually increased over the course of 4 hrs under excitation of 350 nm. Also, it is demonstrated the high photostability of C-paints for 7 days, that is PL properties of C-paints-4 sample (x 50 dilution) remains unchanged after storing at least for one month in air at room temperature. The quantum yield of C-paints was increased from ~1% to ~14% with increasing ultrasound irradiation time. On the basis of equation (see Methods) it can be calculated the quantum yield of C-paints. The quantum yield (QY) based on the equation (1) on these C-paints with tubular form is summarized in Supplementary Table 3. The strongest fluorescent emission with the enhanced maximum quantum yield of ~14% was observed at 370 nm and centered at 450 nm. In the CDs’s formation process, fluorescent C-paints become softer and stickier properties than pure PEG. Ultrasound irradiation at room temperature with no additional solvent removes residues from PEG polymer and endows C-paints with adhesive characteristics, which leads to C-paints attachable to various substrates. This sticky effect is more pronounced to the naked eye when C-paints are “spread” on aluminum foil or paper (Fig. 1c,d).

### Biocompatibility of C-paints

**Figure 2:**
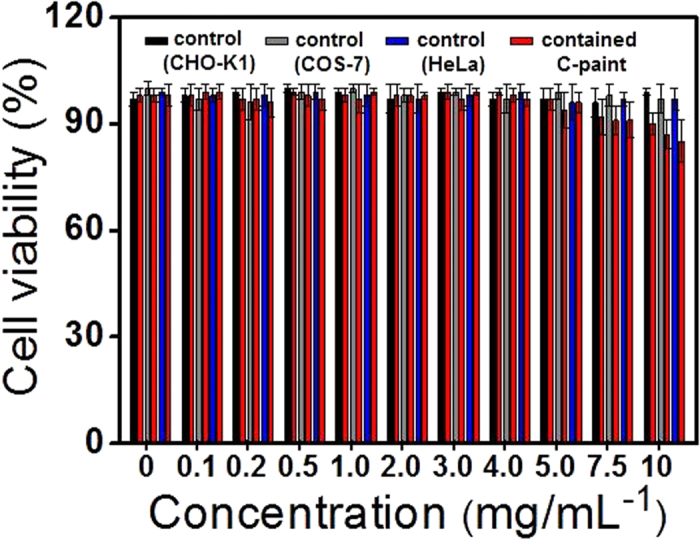
Effect of C-paints on the cell viability. Cytotoxicity of C-paints was analyzed by 3-[4,5-dimethylthiazol-2-yl]-2,5 diphenyl tetrazolium bromide (MTT) assay using the CHO-K1 (ovary; Chinese hamster), COS-7 (kidney, African green monkey) and HeLa (cervical cancer cells, human) cell lines. The cells were treated with various concentrations of C-paints (0 to 10.0 mg/mL).

An important characteristic of fluorescent materials with respect to biological and environmental applications is biocompatibility^23^,^24^. Its cytotoxicity was evaluated by 3-[4,5-dimethylthiazol-2-yl]-2,5 diphenyl tetrazolium bromide (MTT) assay using the CHO-K1 (ovary, Chinese hamster), COS-7 (kidney, African green monkey) and HeLa (cervical cancer cells, human) cell lines. Cells with C-paints concentrations ranging from 0 to 10 mg/mL were incubated for 24 hrs^23^. As shown in Fig. 2, the C-paint showed no inherent cytotoxicity to the CHO-K1, COS-7 or HeLa cells (cellular viability: 97% ± 3% for CHO-K1, 94% ± 5% for COS-7, and 96% ± 3% for HeLa cells) when the amount of C-paints less than 5 mg/mL was used. At higher concentration of C-paints (5 mg/mL), the cell viability was over 95%, indicating good biocompatibility with low- or non-cytotoxicity. Beyond that concentration, the cell viability was slowly decreased in a concentration-dependent manner. Further cytotoxicity testing in animal cells is carefully considered to ensure the environmental safety of C-paints.

### Photocatalytic performances under UV- and visible light

Photocatalysis with sun-light has the potential to be a valuable technology for degradation of harmful organic compounds. For photocatalytic applications, the fraction of UV-light available is very low; therefore, visible light-responsive photocatalysts or additional light sources are needed. As an alternative offer, the use of C-paints as a fluorescent source, for example, can increase the photodegradation activity of TiO_2_ NPs. Based on the FT-IR spectra obtained in the present study, the C-paints exhibited very good solubility in water, due to the presence, in the aqueous photocatalytic systems, of abundant hydrophilic groups that can freely disperse in water for reception and delivery of outer UV- and visible-light.

**Figure 3:**
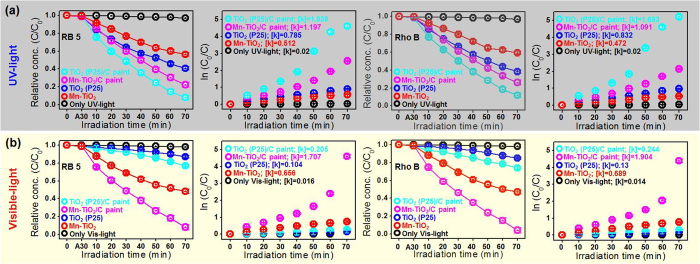
Photocatalytic performances. Relationship between RB5 and Rho B concentration and reaction time (min) for different catalysts (A30: adsorption/desorption equilibrium for 30 min): Mn-TiO_2_, TiO_2_ (P25), Mn-TiO_2_/C-paints and TiO_2_ (P25)/C-paints under (**a**) UV- and (**b**) visible-light irradiation.

The photocatalytic activity and stability of the C-paints, UV-responsive commercial TiO_2_ (P25) and visible-light-responsive 1%-Mn-doped TiO_2_ (Mn-TiO_2_) NPs were evaluated for the degradation of the organic dyes reactive black 5 (RB5) and rhodamine B (Rho B) under UV- and visible-light irradiation^30^. Prior to irradiation, the mixed solution of C-paints, TiO_2_ NPs and organic dyes in aqueous solution was stirred in the dark for 30 min to confirm the adsorption/desorption equilibrium condition (A30). Compared with bare TiO_2_ NPs (commercial P25 and Mn-TiO_2_), TiO_2_ NPs with C-paints exhibited enhanced photocatalytic efficiencies. Figure 3 indicates that the rates of photodegradation of organic dyes for TiO_2_ NPs with C-paints were two-fold higher than for pure TiO_2_ (P25) NPs and three-fold higher than for Mn-TiO_2_ NPs under exciting irradiation. These results show that C-paints can remarkably improve photocatalytic efficiency in the bulk solution. Our finding indicates that the degradation rate was gradually increased with increasing content of C-paints or TiO_2_ NPs in comparison to the control samples (Supplementary Fig. 7).

**Figure 4:**
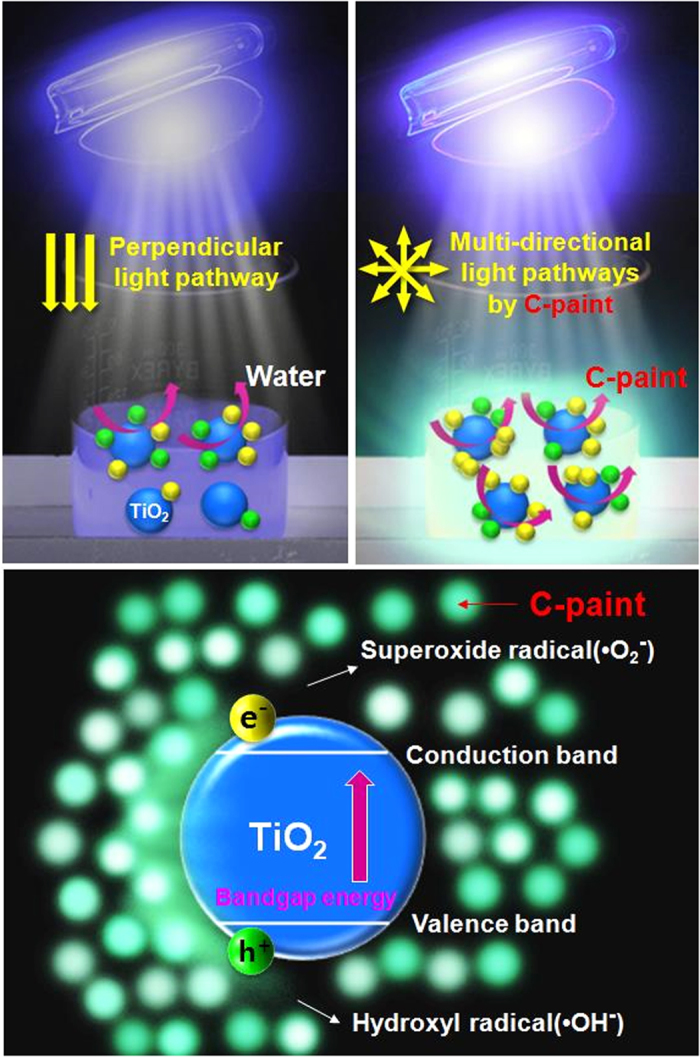
Schematic diagram of photoresponse of C-paint in photocatalytic performance. The photodegradation of RB5 and Rho B in the presence of C-paint under UV (left-top panel) and visible (right-top panel) light, where the enlarged photograph in the bottom showed partial mechanism of photocatalytic activity by C-paints. The schematic graphic was done by Miss So Young Park.

In photocatalytic process of TiO_2_ NPs, C-paints can, as shown in Fig. 4, enhance the amount of light available for TiO_2_ NPs, thereby accelerating photocatalytic TiO_2_ reaction. First, in the shallow region of the dye solution, TiO_2_ NPs exhibited effective photocatalytic activity under close-by lamp irradiation, as the transparent C-paints facilitated the absorption of light into the TiO_2_ NPs NPs. As the light source proceeded towards the perpendicular direction, the light-absorption distance gradually decreased, showing the lowest light absorption at the bottom of the dye solution. However, when C-paints are illuminated, they absorb UV- and visible-light and then emit UV- and visible-light energies, which in turn excites in TiO_2_ and Mn-TiO_2_ NPs again. This is expected to enhance the photo-response of TiO_2_ NPs to form electron-hole pairs. Certainly, C-paints are expected to be a unique material for enabling immediate delivery of light into the surrounding microenvironments of Mn-TiO_2_ NPs either isotropically or multi-directionally. To further demonstrate the photocatalytic stability of the C-paints, recycling tests of RB 5 degradation with TiO_2_ (P25) were performed. As shown in Supplementary Fig. 8, after 10 recycles for the degradation of RB 5, the photocatalytic activity of the C-paints with TiO_2_ system presents about 15% loss. It is associated that loss of TiO_2_ NPs was occurred during washing and drying for reuse. In a particular photocatalytic process, the separation of small-sized TiO_2_ NPs from suspended solution could be a hard work for the purpose in recycling uses^30^.

## Discussion

The concept of C-paints as multi-directional light sources has been demonstrated by using TiO_2_ and Mn-TiO_2_ NPs as an photocatalytic system for simultaneous light adsorption and delivery. The fabricated C-paints with TiO_2_ NPs samples showed much increased photodegradation efficiency. C-paint enhances the UV- and visible light absorbance and regenerate the UV- and visible light for activating TiO_2_ NPs. These results open new perspectives for fluid based mobile light sources. Importantly, C-paints showed high quantum yields (~14%), water solubility and very low cytotoxicity against CHO-K1, COS-7 and HeLa cells. for environmental application.

In summary, treating bare PEG polymer under ultrasound irradiation was proven to be a facile method for large-scale preparation of C-paints. These C-paints are easy to produce at room temperature and can be excited by UV- as well as visible-light. We further demonstrated the use of such C-paints media as high-fluorescent and water-soluble media for an innovative photocatalytic system, finding that designed complex photocatalysts (C-paints/TiO_2_ NPs) efficiently enhanced photocatalytic efficiencies. In this system, C-paints as mobile light sources have obvious advantages in adjusting the decomposition of organic materials by the emitting fluorescence throughout the surface of TiO_2_ NPs in water, which increased light-absorbing surface area of TiO_2_ NPs. Fluorescent C-paints as advanced concept of fluid type light are expected to be utilized effectively and widely in biological processes and flexible optoelectronics.

## Methods

### Fabrication of C-paints

Polyethylene glycol (PEG) (average Mn = 300, Sigma-Aldrich, MO, USA) as a biocompatible non-conjugated polymer was used as CDs source. For the one-step synthesis of C-paints, 40 mL PEG was placed in a glass bottle (57 mm in width × 108 mm in height) as a carbon source was treated with ultrasound irradiation at room temperature temperature (frequency = 40 kHz) for 1, 2, 3 and 4 hrs, respectively. The whole experiment was worked on without additional solvents. As the ultrasound irradiation time increased, the solution changed from colorless to yellow brown. At last, 40 mL brown fluid containing carbon nanoparticles was obtained and named C-paint-n (where n indicates synthesis time). We determine the synthetic conditions (1–4 hrs) under the optimized non-toxic state (<5 hrs; < 5 mg/ml), because we attempted to improve eco-friendly photocatalytic efficiency using C-paints.

### Sample characterization

The morphological structure and size of the C-paints was analyzed by high-resolution transmittance electron microscopy (HRTEM) and atomic force microscopy (AFM, VEECO Instrument, USA). The HRTEM specimens were prepared by drop-casting 10 μL of the C-paints solution on a 300 mesh carbon-coated copper TEM grid with a carbon film, followed by drying at room temperature. For the AFM analysis, 100 mL of the C-paints was placed on a silicon wafer. The wafer was air-dried for 24 hrs, and the remaining solution was dispersed using an air gun. In a corresponding particle-size-distribution histogram, the C-paints were plotted, based on a nanoparticle count. UV/Vis absorption spectra were recorded by a UV-Vis-NIR spectrophotometer (Varian, Cary 5000, Australia). Photoluminescence (PL) spectra were recorded using a UV transilluminator (DUT-260; Core Bio System, Korea) to measure the optical properties of C-paints. The excitation wavelengths were 290–570 nm. The internal quantum yield was measured with the Quantum Yield System (K-MAC, Fluoro-Q2100) at 370 nm excitation by the equation:


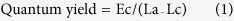


where Ec is the emission produced by direct excitation light, La is the total amount of excitation light and Lc is the amount of light after direct excitation^31^. The reference solution used was water. Transmission Fourier transform infrared (FT-IR) spectra were acquired using a JASCO FTIR 470. Each spectrum was recorded from 4000 to 900 cm^−1^ in 12 scans at a resolution of 4 cm^−1^. High-resolution X-ray photoelectron spectroscopy (HR-XPS) was carried out using monochromatic Al Kα X-ray radiation (hν = 1486.6 eV) with a power of 120 W (Kratos Analytical, AXIS Nova, UK) to investigate the surface properties of the samples. The shift in the binding energy due to relative surface charging was corrected using the C1s level at 284.6 eV as an internal standard. Depth-profiling XPS with Ar^+^ ion bombardment was performed.

### Biocompatibility

CHO-K1, COS-7, and HeLa cells were cultured with Dulbecco’s Modified Eagle’s Medium (DMEM) and Roswell Park Memorial Institute medium (RPMI-1640) supplemented with 10% heat-inactivated FBS (Fetal bovine serum) and 1% antibiotics^23^,^24^. The cells were grown in a humidified incubator at 37^o^C with 5.0% CO_2_. Cells were seeded at a density of 7 × 10^3^ cells per well. Three cells were seeded in a 96-well plate. After overnight culture, different concentrations of C-paints were added to a culture medium and incubated under normal cell culture conditions. Then, 20 L of a 0.2 mg/mL (3-(4,5-dimethylthiazol-2-yl)-5-(3-carboxymethoxyphenyl)-2-(4-sulfophenyl)-2 H-tetrazolium) (MTS) solution in DMEM and/or RPMI-1640 was added to each well and incubated at 37 °C for 2 hrs. Finally, the optical density was measured at 490 nm with an absorbance microplate reader (EMax microplate reader, Bucher Biotec AG, Basel, Switzerland).

### Photocatalytic properties

The rhodamine B (Rho B; 3 mg/L, pH 5.5, Sigma-Aldrich, USA) and reactive black 5 (RB 5; 3 mg/L, pH 6.67, Sigma-Aldrich, USA) solutions were placed in test reactor, and the dye was adsorbed in C-paints under the dark for 30 min (A30). After the adsorption process of the dye was completed, C-paints (0.5–5.0 mg/mL) were dissolved in dye solution and photocatalyst samples (TiO_2_ and Mn-TiO_2_ NPs) were added to the final solution. The photocatalytic degradation of the Rho B or RB 5 solution with catalyst samples (0.5 g/L) and C-paints (0.5–5.0 mg/mL) was carried out under UV- (source: 4 W, 365 nm, VSLAB VL-4CL, Korea) and visible-light (source: 150 W Xe lamp, λ > 420 nm, SCHOTT, USA) irradiation^32^,^33^ and the absorbance of the solutions was measured using a UV-Vis-NIR spectrophotometer (Varian, Cary 5000, Australia) in the wavelength region of 200–800 nm. The post-photo-irradiation concentrations of RB 5 and Rho B in the filtered supernatant solutions were measured from the absorbance peak intensities of the solutions at 598 and 555 nm, respectively^33^,^34^. The changes in the dye-solution concentration [ln(C_0_/C) = *k*t, where *k* is the apparent reaction rate constant, and C_0_ and C are the initial and reaction concentrations at time = 0 and time = t, respectively, of Rho B] also were investigated. To investigate the repeatability of the C-paints complex photocatalytic system, photocatalytic experiments were repeated. After reactions, the TiO_2_ NPs were collected by centrifugation (6000 rpm, 10 min). The separated TiO_2_ NPs was performed washing with distilled (DI) water and drying in an oven (65 °C) for 3 hrs after every cycle. The treated TiO_2_ NPs was reused in the photocatalytic reaction for 10 times under the identical C-paints and light irradiation conditions.

## Additional Information

**How to cite this article**: Young Park, S. *et al.* Eco-friendly carbon-nanodot-based fluorescent paints for advanced photocatalytic systems. *Sci. Rep.* **5**, 12420; doi: 10.1038/srep12420 (2015).

## Supplementary Material

Supplementary Information
