# Supplementary material for: Eco-friendly carbon-nanodot-based fluorescent paints for advanced photocatalytic systems
Source: Sci Rep. 2015 Jul 23;5:12420. doi: 10.1038/srep12420 (PMC5378878; doi:10.1038/srep12420)
Supplement: Supplementary Information [file srep12420-s1.doc]

**Supplementary Information**

**Eco-friendly carbon-nanodot-based fluorescent paints for advanced photocatalytic systems**

**So Young Park^1,*^, Hyun Uk Lee^1,*^, Young-Chul Lee^2,*^, Saehae Choi^3^, Dae Hyun Cho^3^, Hee Sik Kim^3^, Sunghee Bang^4^, Soonjoo Seo^1^, Soon Chang Lee^5^, Jonghan Won^1^, Byung-Chul Son^6^, Mino Yang^7^ & Jouhahn Lee^1,*^**

^1^Division of Materials Science, Korea Basic Science Institute (KBSI), Daejeon 305-333, Republic of Korea. ^2^Department of BioNano Technology, Gachon University, Gyeonggi-do 461-701, Republic of Korea. ^3^Environmental Biotechnology Research Center, Korea Research Institute of Bioscience and Biotechnology (KRIBB), Daejeon 305-806, Republic of Korea. ^4^Department of Engineering (Nanotechnology Engineering), University of Waterloo 200 University Avenue West, Waterloo, Ontario, N2L 3G1, Canada. ^5^Department of Applied Chemistry and Biological Engineering, Chungnam National University, Daejeon 305-764, Republic of Korea. ^6^Korea Advanced Institute of Science and Technology (KAIST), Research Analysis Center, Daejeon 305-701, Republic of Korea. ^7^Division of Analytical Research, Korea Basic Science Institute (KBSI), Gangneung 200-701, Republic of Korea.

* These authors contributed equally to this work.

Correspondence and requests for materials should be addressed to H.U.L (email: leeho@kbsi.re.kr), Y.-C.L (email: dreamdbs@gachon.ac.kr) and J.L (email: jouhahn@kbsi.re.kr)

**Supplementary Table 1 |** XPS analysis results of C-paints.

| **Name** | **Star BE** | **Peak BE** | **End BE** | **Height CPS** | **FWHM eV** | **Atomic %** |
| --- | --- | --- | --- | --- | --- | --- |
| **C1s** | 291.18 | 285.98 | 280.83 | 19416.26 | 1.072 | 68.04 |
| **O1s** | 537.18 | 532.37 | 528.13 | 21653.08 | 1.224 | 31.96 |

**Supplementary Table 2 | The position and area of the deconvoluted C1s peaks for C-paints.**

|  | **-C-C or –C-H** | **-C-O(H)** | **C-O-C=O** |
| --- | --- | --- | --- |
| **Position** | 284.6 | 286.2 | 287.3 |
| **Area** | 1529.3033 | 15835.9844 | 2174.5913 |

**Supplementary Table 3 │Quantum yield measurements of C-paints**

| **Synthesis time (hr) of C-paints** | **La** | **Lc** | **Ec** | **Quantum yield (QY)** |
| --- | --- | --- | --- | --- |
| **0** | 59,120 | 58,299 | 788 | 0.95 |
| **1** | 58,954 | 58,299 | 2,610 | 3.98 |
| **2** | 56,658 | 55,438 | 8,504 | 6.97 |
| **3** | 55,840 | 54,321 | 12,076 | 7.95 |
| **4** | 56,840 | 55,858 | 14,030 | 14.28 |


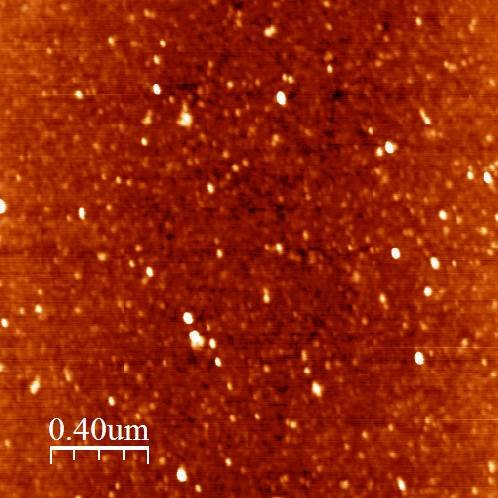


**Supplementary Figure 1 | AFM characterization of the C-paints.**

AFM images shows the C-paints are spherical and well dispersed with the size of 1-8 nm in diameter and no CD aggregation is observed.


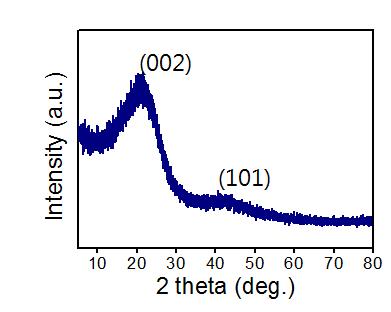


**Supplementary Figure 2 | XRD pattern of C-paints.**


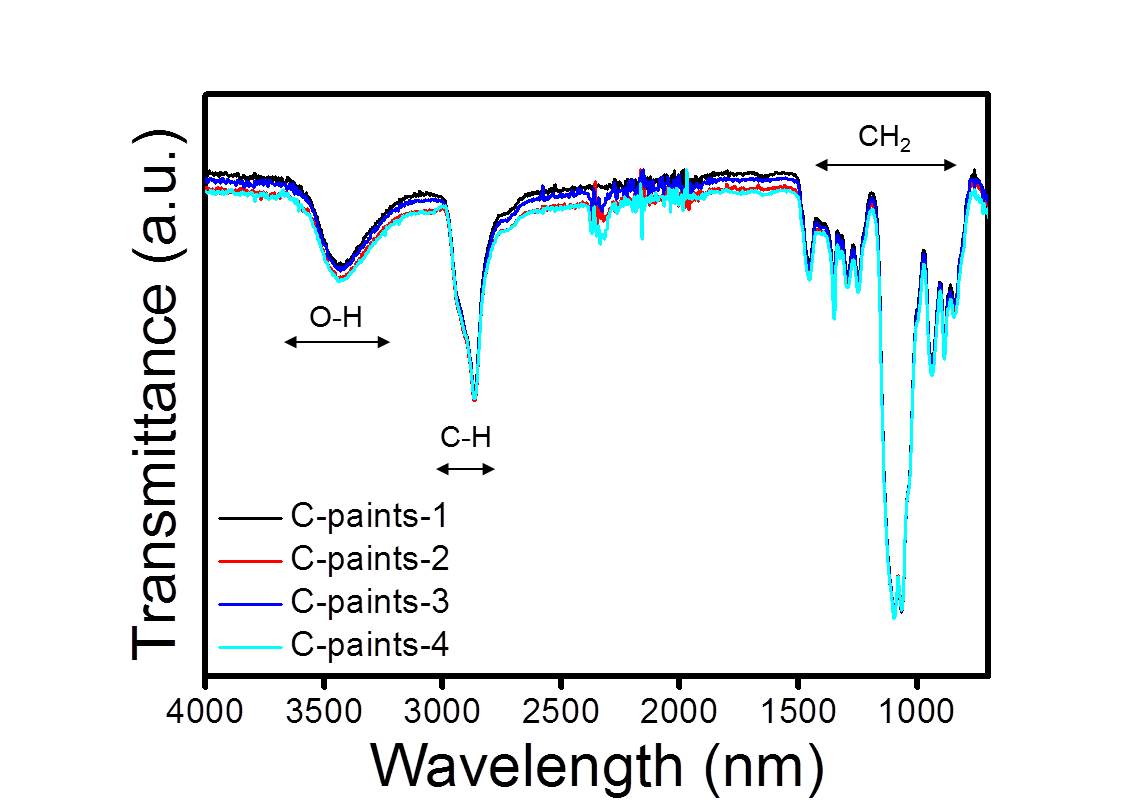


**Supplementary Figure 3 |** **FT-IR spectrum of C-paints.**

The chemical bonding states of C-paints shows oxygen groups without any further surface passivation.


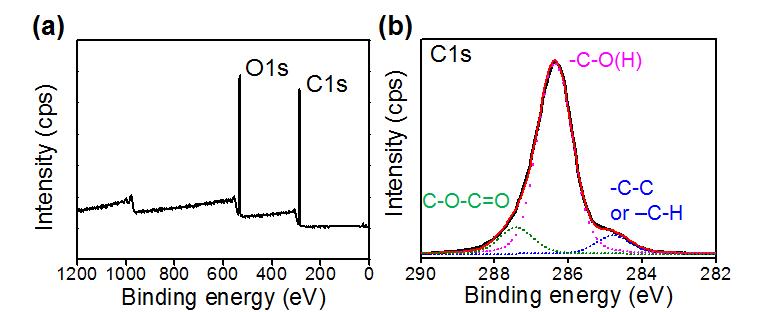


**Supplementary Figure 4 | (a) Wide XPS survey and (b) high-resolution XPS C1s spectra of C-paints.**

**
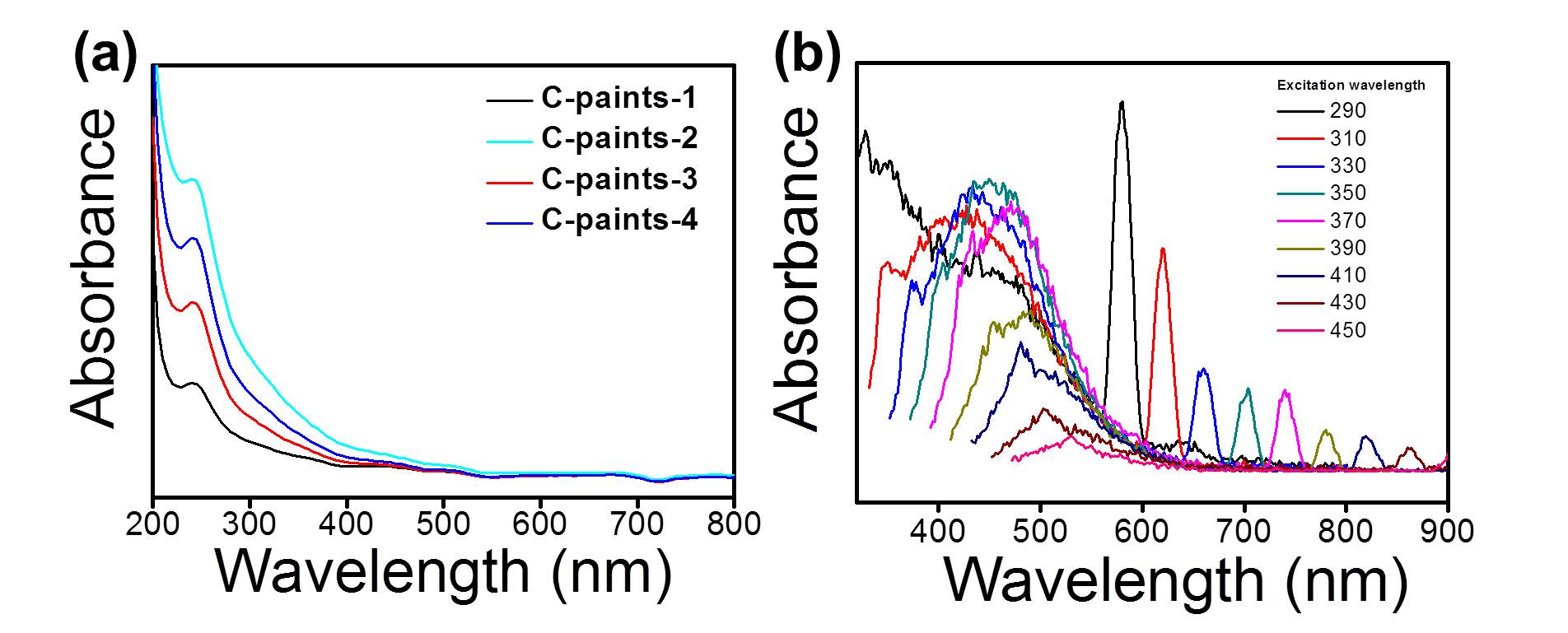
**

**Supplementary Figure 5 | Optical properties of C-paints.**

(a) UV-Vis and (b) photoluminescence spectra of C-paints.


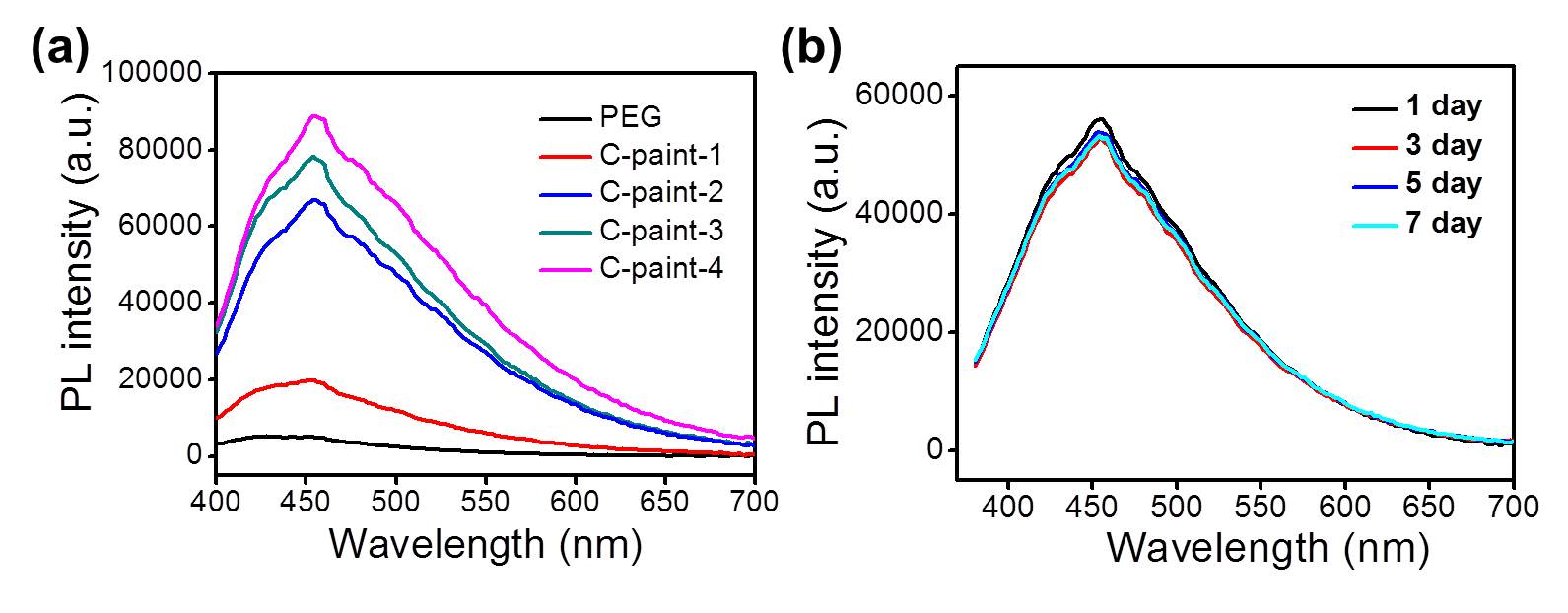


**Supplementary Figure 6 | (a) PL emission spectra of C-paints at different synthesis times (hr) and (b) photostability of C-paints for 7 days.**

The PL spectra of the C-paints were gradually increased over the course of 4 hrs with synthesis times and stable intensity was observed for 7 days.


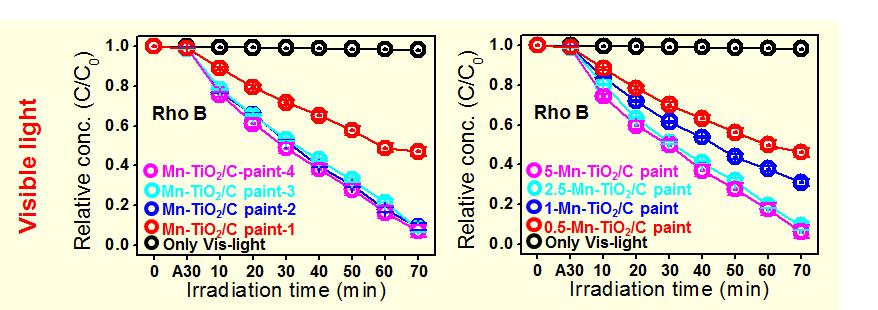


**Supplementary Figure 7 | Photocatalytic activities using Mn-TiO_2_ with C-paints.**

Photocatalytic analysis was obtained with different types of (a) synthesis time of C-paints and (b) concentration of Mn-TiO_2_ under visible-light.


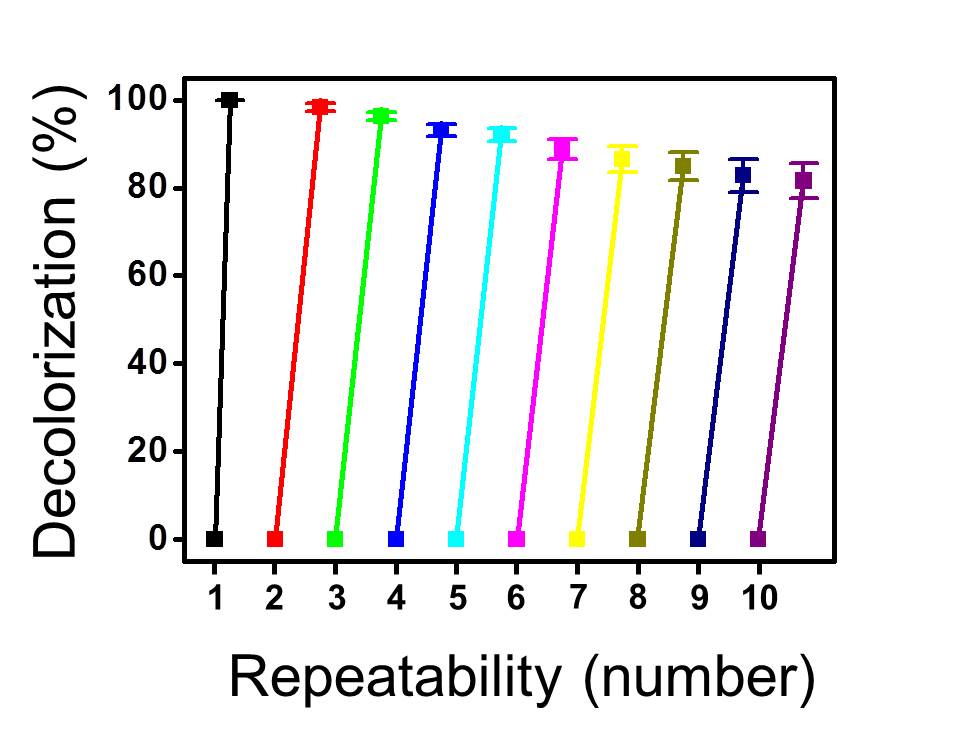


**Supplementary Figure 8│Recycling properties in photocatalytic performances.**

The photocatalytic stability of C-paints with TiO_2_ (P25) in recycling reactions.
